# Evaluation of the Cause of Dental Treatment in Children under General Anesthesia from the Perspective of Pediatric Dentists and Postgraduate Students and Parents of Children under General Anesthesia at the Dentistry School of Tehran University of Medical Science

**DOI:** 10.1155/2022/6934016

**Published:** 2022-05-27

**Authors:** Alireza Heidari, Mehdi Shahrabi, Zohre Salehi, Fateme Kalantari, Zahra Salehi

**Affiliations:** ^1^Department of Pediatric Dentistry, School of Dentistry, Tehran University of Medical Sciences, Tehran, Iran; ^2^School of Medicine, Isfahan University of Medical Sciences, Isfahan, Iran

## Abstract

**Background:**

Nowadays, dental treatment under general anesthesia is accepted as a treatment method in children in which other common methods do not allow to achieve acceptable results. The benefits of general anesthesia in pediatric dental treatment are that the treatment is completed in one session, the person is relieved of pain, and most importantly, the child does not need to cooperate. However, it is important to determine the exact dental reasons for children under general anesthesia.

**Objective:**

In this study, the cause of dental treatment under general anesthesia in children was examined from the perspective of specialists, assistants, and parents of children under general anesthesia.

**Methods:**

In this descriptive-analytical cross-sectional study, participants were selected from among the parents of children under general anesthesia for dental treatment and pediatric dentists and assistants in a purposeful manner. After the items were prepared by the professors, the prepared checklist was read to the people. After completing the checklist, the data were entered into SPSS software version 20 and analyzed using descriptive statistics such as frequency determination, mean, and statistical rests.

**Results:**

According to the consensus of parents and experts, the main reasons for general anesthesia for children to perform their dental operations were: young age, lack of cooperation during previous dental treatment, a large number of dental treatments, systemic disease of the child, unwillingness of parents to perform behavioral control procedures, higher quality work under anesthesia, mental and physical behavioral problems, and reduced number of treatment sessions.

**Conclusion:**

Therefore, reasons such as young age, lack of cooperation, and a large number of dentist's work are among the most important factors that lead to the decision to perform general anesthesia in children. In light of this decision, the consequences and disadvantages/advantages of this method are important and therefore more research should be done on this issue.

## 1. Introduction

Oral health of children at the age of deciduous teeth and its promotion is one of the issues that is less considered by families, doctors, and paediatricians [1]. Many oral health problems, including dental caries, begin in childhood and can have a significant impact on a child's development, general health, and quality of life [2–4]. On the other hand, premature loss of deciduous teeth due to extensive caries causes problems with lack of space, malocclusion, and problems in the eruption of permanent teeth [5].

With the increase of parents' awareness of dental treatments, attention to pediatric dental treatment and early dental procedures has increased. But there is a problem with children's behavior control and their fear of dental treatment, especially extensive treatments [6]. The most important causes of fear in children are general fear, the child's age, and parents' fear of medical teeth [7].

Lack of cooperation and consequently physical restraint of some children, in addition to reducing the accuracy and quality of work, will create unpleasant psychological results for them[8]. In addition, there are children with special medical needs such as communication disabilities, mental disabilities, physical limitations, mobility limitations, behavioral disorders, and chronic medical conditions who require special therapeutic interactions in behavioral control techniques to perform dental work [9]. Practical techniques include desensitizing patients, prescribing antianxiety drugs, immobilizing limbs, sedating the patient, and finally general anesthesia [10].

According to the AAPD protocol, anesthesia prescriptions are:

1. Patients with specific mental, physical, or medical problems
2. In patients on whom local anesthesia is unusable due to acute infection, anatomical variations, or allergies
3. Emotionally and physiologically immature noncooperative patients
4. Patients need comprehensive and immediate treatment that cannot be completely cured by other means [11–13]

Dentistry under anesthesia has a positive psychological effect on treated children and strengthens the positive attitude of parents and children towards oral health [14]. It seems that after this type of treatment, the child can make some changes in his behavior and life, such as increasing the number of times he brushes and reducing the consumption of high-sugar foods [15].

Despite the high risk of side effects in general anesthesia, this method is known to be a safe and secure method in hospitals and operating rooms [16]. The use of this method by pediatric dentists is increasing and also its popularity among parents has increased from the last 30 years until today [17].

Numerous studies show that the effects of anesthesia in children for exposure to general anesthesia before the age of 4 increase the risk of cognitive problems and ADHD in children [18]. In this regard, the number and frequency of anesthesia is also important. Surgery lasting less for than 2 hours does not pose any risk, but surgeries longer than 3 hours increase the likelihood of learning disabilities [19]. General anesthesia should not be performed for some systemic diseases. From the parents' point of view, factors such as the effects of anesthesia and fear of anesthesia are among the disadvantages of general anesthesia.

Treatment under general anesthesia has several advantages, including the efficiency and convenience of treatment, extensive treatment in one session, and the absence of unpleasant memories after dental treatment. However, this type of treatment is also associated with problems such as stress of the child and parents during treatment and related costs [20].

General anesthesia means reducing the patient's level of consciousness to the point that the senses (especially the sensation of pain) are inactive. This type of anesthesia is usually done by injecting anesthetics into a vein or inhaling the anesthetic through a mask. This will be done in the operating room under the supervision of an anesthesiologist. General anesthesia is divided into 4 stages: induction, excitement, surgical anesthesia, and overdose [7].

General anesthesia has 5 goals: analgesia, memory loss and surgery, immobility, loss of consciousness, and muscle relaxation [7].

Inhalation anesthesia methods include open, semiopen, semiclosed, and closed systems. The semiclosed system is often used in new anesthetics. Its benefits include reduced temperature drop and body water vapor, saving gas consumption, and reducing environmental pollution. Exhausting anesthetics available include nitrous oxide, halothane, influenza, isoflurane, desflurane, and sevoflurane. For decades, halothane has been very popular as a drug to induce anesthesia in children. Recently, sevoflurane has been introduced and has a lower blood/gas dissociation rate than halothane. As a result, sevoflurane has become the drug of choice for inhalation anesthesia [21].

In this study, the aim was to determine the causes of dental treatments under general anesthesia in children from the perspective of pediatric dentists and postgraduate students and parents of children under anesthesia in the pediatric dentistry department of Tehran University of Medical Sciences in 2020. Null hypotheses: according to pediatric dentists, postgraduate students and parents, the reason for performing dental treatments under general anesthesia in children is probably due to the children's fear.

## 2. Methods and Materials

This study was a descriptive-analytical cross-sectional study with the code of ethics of IR TUMS Dentistry. REC.13980040 in which the participants were the parents of children under general anesthesia, pediatric dentists, and postgraduate students of pediatric dentistry.

According to the results of the study of Eshghi et al. [22] and considering the target width: 0.12, *p*=0.75, *a* = 0.5, the minimum required sample size was estimated at 215 samples.

### 2.1. Inclusion Criteria

1. Parents of children who underwent general anesthesia for dental treatment at the School of Dentistry of Tehran University of Medical Sciences.
2. Pediatric dentists
3. Postgraduate students of pediatric dentistry.

### 2.2. Exclusion Criteria

1. Withdrawal from cooperation.

The descriptive-analytical cross-sectional study was performed by a checklist. The items of this checklist were determined by the brainstorming of the pediatric professors of Tehran University of Medical Sciences who participated in the operating room under anesthesia. After reviewing and collecting the items, a checklist was prepared by the professors, and the validity and reliability of the available items were evaluated from the perspective of experts and their validity from the perspective of parents and was made available to individuals.

Participants in this study consisted of two groups and were selected from the parents of children who underwent general anesthesia for dental treatment and pediatric dentists and postgraduate students in pediatric treatment centers of the School of Dentistry of Tehran University of Medical Sciences. In this study, assistants and specialists were placed in a group. The parents were selected randomly and the study population in the group of specialists and assistants was all specialists and assistants working in the pediatric center of the School of Dentistry of Tehran University of Medical Sciences. Randomization was done using a table of random numbers. The checklists were collected in about 3 months. The parent checklist included two sections of demographic information and 9 reasons for choosing anesthesia treatment.

Demographic information includes the child's age, sex, parent's education level, parent's occupation, dental work history, home address, number of households, total number of anesthesia times and 9 reasons for prescribing treatment under anesthesia, including young age, lack of cooperation during previous dental treatment, a large number of dental work, systemic disease of the child, unwillingness of parents to perform behavioral control methods, higher quality work under anesthesia, mental problems, behavioral physics, reducing the number of treatment sessions, receiving more money by the dentist. Checklists were given to specialists after anesthesia treatment and for parents in the parents' waiting room.

The present checklists were provided to the participants with full satisfaction and knowledge of the contents of the study, then the desired information was collected and the data were analyzed by regression model.

Statistical methods of data analysis: Then, after completing the checklists, the data were entered into SPSS statistical software version 20 and analyzed from the perspective of parents and experts using descriptive statistics such as determining the frequency and determining the percentage of overlap of all items mentioned in the checklist.

## 3. Results

According to the demographic information, the age range of children in the study was between 2 and 11 years-old, and the average age was 4 years old. About 52% of patients were boys and about 48% were girls. Demographic results showed that 18.5, 25, 34.3, 14.8, and 7.4% of paternal parents have undergraduate, diploma, bachelor, masters, and doctorate education, respectively. About mother's education 13, 38.9, 35.2, 9.3, and 3.7% have undergraduate, diploma, bachelor, master, and doctoral education, respectively. About the father's job 2.8, 50, and 47.2%, respectively, were unemployed, self-employed, and employed 72.2, 12, and 15.7% of mothers are unemployed, self-employed, and employed, respectively. About 23% of people live in other cities and 77% live in Tehran. About 3.7%, 50%, 38%, and 8.3% of households are 2-person, 3-person, 4-person, and 5-person, respectively. 77.8%, 16.7%, 3.7%, and 1.9% of pediatric patients have no history of anesthesia, 1 time, 2 times, and 3 times, respectively.

The most important reasons for anesthesia for pediatric dentistry from the parents' point of view include three factors: young age (74.1%), lack of cooperation during dental treatment in previous (47.2%), and a large number of dental work (35.2%) (Table 1).

Also, the most important reasons for performing anesthesia for pediatric dentistry according to experts included three factors: young age (64.8%), lack of cooperation during previous dental treatment (49.1%), and a large number of dental work (63.9%) (Table 2). It should be noted that parents and professionals could cite more than one factor for the child's anesthesia.

The results of the overlap of the responses of specialists and parents of children under anesthesia about the cause of dental treatment under anesthesia in a child are given in Table 3. According to the consensus of parents and experts, the main reasons for general anesthesia of children for their dental treatment were: young age, lack of cooperation during dental treatment in previous times, a large number of dental work, systemic disease of the child, parents' unwillingness to do behavioral control methods, higher quality work under anesthesia, mental and physical behavioral problems, and reduction in the number of treatment sessions.

## 4. Discussion

One of the difficulties for families and the dental community, especially dentists who deal with children, is children's fear of dental treatment [23]. Research has shown various variables for anxiety and fear of dentistry in children, some of which include maternal anxiety, awareness of dental problems, past dental experiences, unfamiliar sounds, and strange and unfamiliar odors [24]. Anxiety in small degrees can lead to irregular visits and a lack of follow-up treatment. On a larger scale, this anxiety causes many problems such as sleep disorders, negative thoughts, low self-esteem, and even depression [25].

There are several techniques for controlling dental anxiety, including the use of sedatives and hypnotics, behavioral control methods, and a combination of both [25]. All of the above methods, although helpful in many cases, also have their drawbacks. For example, behavioral control methods used at high levels of anxiety cannot be responsive. Also, the use of drugs that slow breathing and cause nausea reflex dysfunction can potentially have risks. In some cases, due to the wide range of treatments needed by the child and the child's lack of cooperation and behavioral control problems, not all of the above methods are effective and dentists are forced to use general anesthesia to treat the child [26].

The results of this study showed that the main reasons for general anesthesia in children for dental work are: young age, lack of cooperation during the previous dental treatment, a large number of dental work, reduction of the number of treatment sessions, and higher quality of work under anesthesia. Consistent with these results, Escanilla-Casal et al. showed that although the amount of complications after surgery under general anesthesia may be high, it is sometimes recommended by parents due to certain conditions (such as the child's lack of serious cooperation) [27].

Pohl and colleagues found that failure to cooperate in dental treatment required treatment under general anesthesia [28], and Nunn and colleagues also suggested the need for extensive treatment, behavioral control problems for children with medical hazards, young children or disabilities, high anxiety, and long distances from dental treatment facilities as definitive prescriptions for treatment under general anesthesia [29]. Gharavi and Soltani in their study revealed that general anesthesia is recommended by dentists in cases of noncooperation or the presence of disabilities with acceptable results and low and preventable complications [30]. These results were consistent with the studies of Malden, Thomson [31], and Sari et al. [32].

In the results of our study, 57.4% of parents and specialists shared the opinion that the young age of the patient is the cause of anesthesia. According to previous studies, such as the study of Eshghi et al., Tyrer et al., Nunn et al., and Malden and Thomson, young age is one of the most common causes for dentistry in children under general anesthesia [18, 22, 29, 31].

Another dental cause of children under anesthesia, in addition to young age, is a lack of cooperation for dental treatments, which in our study in more than 30% of patients was the common opinion of parents and specialists. Other studies such as Eshghi et al., Pohl et al., and Nunn et al. agree with the results of the present study [22, 28, 29].

The large number of dental jobs is not a convincing reason for working under anesthesia, but in situations such as the distance from the parents to the dentist can be one of the causes of anesthesia. For this reason, the common opinion of parents and experts in this case was less than in the other two cases. This is in contrast to the study by Nunn et al., who identified the need for extensive treatment as one of the definitive aspects of treatment under general anesthesia [29].

Contrary to the study of Reich et al., one of the benefits of treatment under general anesthesia is the lack of unpleasant memories after dental treatment. In our study, this option was not selected as the main factor because of the desire of Iranian parents to perform behavioral control methods if the child does not cooperate and avoid general anesthesia as much as possible [33].

In this study, contrary to pediatric dental references, which consider higher quality of work as one of the causes of general anesthesia, in this study, higher quality of anesthesia treatment is not the main reason for performing dental work under anesthesia [21].

In this study, in 6 patients, according to experts, physical, mental, and behavioral problems were the causes of anesthesia, while from the parents' point of view, this factor was not the cause. This could be due to hiding parents from their child's problems or not accepting these problems. In Tyrer, Thomson and Nunn's studies, physical-mental and behavioral problems are also among the causes of dental treatment for children under general anesthesia [18, 29, 31].

Contrary to the study of Nunn et al., long distances from dental treatment sites are suggested as definitive cases of treatment under general anesthesia. In our study about the long distance and the decrease in the number of treatment sessions in 95 patients, according to experts and parents, the main causes are not mentioned, and it seems that in our country this cause is not well accepted among the people [29].

None of the parents and specialists consider the dentist to receive more money as the reason for working under anesthesia, and it can be concluded that dentists only perform dental work under general anesthesia according to the condition of children [29].

## 5. Conclusion

Our results showed that the reasons for performing dental treatments under general anesthesia in children from the point of view of pediatric dentists and postgraduate students and parents of children in the Department of Dentistry of the University of Tehran are as follows: young age, lack of cooperation during dental treatment, a large number of dental treatments, systemic disease of the child, unwillingness of parents to perform behavioral control methods, higher quality of work under anesthesia, mental and physical behavioral problems, and reduced number of treatment sessions.

The highest rate of overlap at a young age, lack of cooperation, a large number of dental treatment that both groups agreed and the highest level of overlap that both groups opposed included higher costs by the dentist and pediatric systemic disease.

The least amount of overlap between experts in favor and parents against was the large number of dental treatments and the quality of dental work.

## Figures and Tables

**Table 1:** Results from the parent's checklist regarding the reasons for general anesthesia of children for dental treatment.

	Variables	Number	Percentage
Parent's checklist	1. Young age	80	74.1
	2. Lack of cooperation during dental treatment in previous	51	47.2
	3. Lots of dental work	38	35.2
	4. Systemic disease of the child	7	6.5
	5. Parents' unwillingness to perform behavioral control methods	10	9.3
	6. Higher quality of work under anesthesia	13	12
	7. Mental, physical, and behavioral problems	5	4.6
	8. Reducing the number of treatment sessions	13	12
	9. Receive more cost by dentist	0	0

**Table 2:** Results from the specialist's checklist regarding the reasons for general anesthesia of children for dental treatment.

	Variables	Number	Percentage
Specialist's checklist	1. Young age	70	64.8
	2. Lack of cooperation during dental treatment in previous	53	49.1
	3. Lots of dental work	69	63.9
	4. Systemic disease of the child	7	6.5
	5. Parents' unwillingness to perform behavioral control methods	15	13.9
	6. Higher quality of work under anesthesia	26	24.1
	7. Mental, physical, and behavioral problems	10	9.3
	8. Reducing the number of treatment sessions	1	0.9
	9. Receive more cost by dentist	0	0

**Table 3:** The extent of overlap between the responses of specialists and parents of children under anesthesia about the cause of dental treatment under anesthesia in a child.

	Common opinion of parents and specialists	Common dissenting opinion of parents and specialists	Parents agree and specialists disagree	Specialists agree and parents disagree
Young age	In 62 patients (57.4%)	In 20 patients (18.5%)	In 18 patients (16.7%)	In 8 patients (7.4%)
Lack of cooperation during dental treatment in previous	In 34 patients (31.5%)	In 37 patients (34.3%)	In 17 patients (15.7%)	In 19 patients (17.6%)
Lots of dental work	In 27 patients (25%)	In 28 patients (25.9%)	In 11 patients (10.2%)	In 42 patients (38.9%)
Systemic disease of the child	In 7 patients (6.5%)	In 101 patients (93.5%)	—	—
Parents' unwillingness to perform behavioral control methods	In 6 patients (5.6%)	In 89 patients (82.4%)	In 4 patients (3.7%)	In 9 patients (8.3%)
Higher quality of work under anesthesia	In 4 patients (3.7%)	In 73 patients (67.6%)	In 9 patients (8.3%)	In 22 patients (20.4%)
Mental, physical, behavioral problems	In 4 patients (3.7%)	In 97 patients (89.8%)	In 1 patients (0.9%)	In 6 patients (5.6%)
Reducing the number of treatment sessions	In 1 patients (0.9%)	In 95 patients (88%)	In 12 patients (11.1%)	—
Receive more cost by dentist	—	In 108 patients (100%)	—	—

## Data Availability

The data are available from the corresponding author on reasonable request.
